# Synergistic cytotoxicity of histone deacetylase and poly-ADP ribose polymerase inhibitors and decitabine in pancreatic cancer cells: Implications for novel therapy

**DOI:** 10.18632/oncotarget.28588

**Published:** 2024-06-03

**Authors:** Benigno C. Valdez, Apostolia M. Tsimberidou, Bin Yuan, Yago Nieto, Mehmet A. Baysal, Abhijit Chakraborty, Clark R. Andersen, Borje S. Andersson

**Affiliations:** ^1^Department of Stem Cell Transplantation and Cellular Therapy, The University of Texas MD Anderson Cancer Center, Houston, TX 77030, USA; ^2^Department of Investigational Cancer Therapeutics, The University of Texas MD Anderson Cancer Center, Houston, TX 77030, USA; ^3^Department of Biostatistics, The University of Texas MD Anderson Cancer Center, Houston, TX 77030, USA

**Keywords:** decitabine, HDAC inhibitors, pancreatic cancer, PARP inhibitors, synergistic cytotoxicity

## Abstract

Histone deacetylase inhibitors (HDACi) can modulate the acetylation status of proteins, influencing the genomic instability exhibited by cancer cells. Poly (ADP ribose) polymerase (PARP) inhibitors (PARPi) have a direct effect on protein poly (ADP-ribosyl)ation, which is important for DNA repair. Decitabine is a nucleoside cytidine analogue, which when phosphorylated gets incorporated into the growing DNA strand, inhibiting methylation and inducing DNA damage by inactivating and trapping DNA methyltransferase on the DNA, thereby activating transcriptionally silenced DNA loci. We explored various combinations of HDACi and PARPi +/− decitabine (hypomethylating agent) in pancreatic cancer cell lines BxPC-3 and PL45 (wild-type BRCA1 and BRCA2) and Capan-1 (mutated BRCA2). The combination of HDACi (panobinostat or vorinostat) with PARPi (talazoparib or olaparib) resulted in synergistic cytotoxicity in all cell lines tested. The addition of decitabine further increased the synergistic cytotoxicity noted with HDACi and PARPi, triggering apoptosis (evidenced by increased cleavage of caspase 3 and PARP1). The 3-drug combination treatments (vorinostat, talazoparib, and decitabine; vorinostat, olaparib, and decitabine; panobinostat, talazoparib, and decitabine; panobinostat, olaparib, and decitabine) induced more DNA damage (increased phosphorylation of histone 2AX) than the individual drugs and impaired the DNA repair pathways (decreased levels of ATM, BRCA1, and ATRX proteins). The 3-drug combinations also altered the epigenetic regulation of gene expression (NuRD complex subunits, reduced levels). This is the first study to demonstrate synergistic interactions between the aforementioned agents in pancreatic cancer cell lines and provides preclinical data to design individualized therapeutic approaches with the potential to improve pancreatic cancer treatment outcomes.

## INTRODUCTION

One of the epigenetic modifications is histone acetylation which is catalyzed by histone acetyltransferases. During this process, the N-terminal tails of histones undergo acetylation by adding acetyl groups to the positively charged lysine residues. This modification reduces the interactions between histones and negatively charged DNA, which results in the relaxation of the chromatin structure. Increased transcriptional activation has been associated with relaxed chromatin [1]. Histone deacetylases (HDACs) reverse this process by removing the acetyl group, leading to a condensed, transcriptionally inactive chromatin. The histone acetylation/deacetylation process induces structural alterations in distant chromosome regions, thereby having a broad impact on gene expression and various cellular processes such as DNA replication and cell division. In particular, HDAC overexpression may down-regulate tumor suppressor genes [2]. Various HDAC inhibitors (HDACis) have been developed to induce gene expression, leading to cell differentiation, cell cycle arrest, and apoptosis [3]. Some HDACis have regulatory approval for the treatment of patients with hematologic malignancies, including vorinostat, romidepsin, panobinostat, and belinostat.

Although HDACis have shown promising results in preclinical studies, their clinical efficacy as monotherapy is limited; however, when combined with other anticancer drugs, enhanced anti-tumor activity has been reported [4]. The diverse impact of HDACis on regulating cellular drug transporters should be considered when planning their use in combination with chemotherapy. For example, in human hematologic cancer cell lines, HDACis have been shown to decrease expression of the MRP1 protein and increase expression of the MDR1 protein [5]. Notably, in patients with previously untreated peripheral T-cell lymphoma, the addition of the HDACi romidepsin to cyclophosphamide, doxorubicin, vincristine, and prednisone (CHOP) did not improve clinical outcomes [6]. The lack of clinical improvement when romidepsin was added to MDR1 ligands (e.g., doxorubicin, vincristine) may be at least partially attributed to the effects of romidepsin on the expressions of MRP1 and MDR1 [6]. The addition of HDACis to chemotherapy may enhance its efficacy by inducing DNA double-strand breaks. Indeed, alterations in chromatin structure induced by HDACis directly trigger activation of the DNA damage response [7].

HDACis can affect the acetylation of proteins involved in different DNA repair mechanisms, thereby influencing the genomic instability displayed by cancer cells. Researchers have extensively studied the effects of HDACis on genomic stability, as well as their interactions with poly (ADP ribose) polymerase (PARP) inhibitors (PARPi), particularly in solid tumors [8–11]. PARPis have a direct effect on protein poly (ADP-ribosyl)ation (PARylation), which is important for DNA repair [12]. PARP enzymes catalyze PARylation and bind to DNA breaks, self-ribosylate, and recruit and PARylate DNA repair proteins [12]. We have previously reported on the synergistic activity of HDACis and PARPis in hematologic malignancies that occurs via enhanced inhibition of protein PARylation and decreased levels and phosphorylation of major proteins involved in DNA repair [13]. Similar synergistic interactions between HDACis and PARPis have been reported in thyroid and breast cancer cells [9, 10]. Published data suggest that the synergism may be partly attributed to blocking chromatin PARylation with histone acetylation [12].

For patients with metastatic pancreatic adenocarcinoma bearing *BRCA1/2* germ-line mutations (*gBRCAm*; *BRCA1* prevalence, 0.3% to 2.3% and *BRCA2* prevalence, 0.7 to 5.7%) [14–16] who achieve clinical response to first-line treatment, the PARPi olaparib is now offered as an alternative maintenance treatment option. In a randomized study (POLO), 154 patients with *gBRCAm* metastatic pancreatic adenocarcinoma treated with olaparib had longer progression-free survival (7.4 months) than those treated with placebo (3.8 months) (hazard ratio = 0.53; *p* = 0.0035) [17]. No statistically significant difference was noted in overall survival [18].

Decitabine (5-aza-2′-deoxycytidine) is a nucleoside cytidine analogue that, when phosphorylated, is incorporated into a growing DNA strand, and inhibits DNA methylation. Decitabine also induces DNA damage by inactivating and trapping DNA methyltransferase on DNA, consequently activating transcriptionally silenced DNA loci [19–21]. KRAS-dependent gene signatures have been reported to be associated with sensitivity to decitabine in selected patients with *KRAS*-mutated pancreatic cancer [22]. Other nucleoside analogues (e.g., gemcitabine) have established antitumor activity in pancreatic cancer.

Therefore, we explored various combinations of HDACis and PARPis, with or without decitabine, in pancreatic cancer cell lines. In this study, we show that HDACis inhibit protein PARylation and exhibit synergistic cytotoxicity with PARPis and a demethylating agent (decitabine). The results provide novel preclinical data that demonstrate synergism between HDACi- and PARPi-mediated inhibition of DNA repair and decitabine in pancreatic cancer and have implications for the exploitation of therapeutic purposes.

## RESULTS

### Sensitivity of pancreatic cancer cell lines to HDACis, PARPis, and decitabine

**Figure 1:**
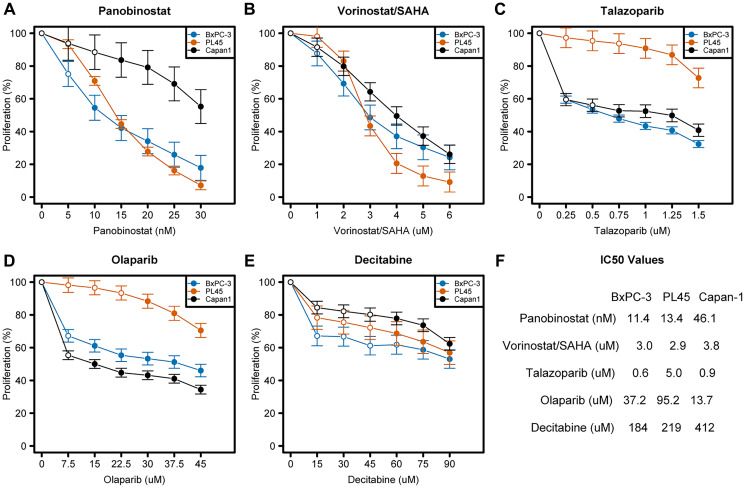
Dose-response curves of various drugs in three pancreatic cancer cell lines. (**A**–**F**) Cells were seeded in 96-well plates overnight and exposed to drugs for 3 days as described in the Materials and Methods. Rate of cell proliferation was determined relative to control by MTT assay. Model-adjusted means are shown with 95% confidence intervals for the non-zero doses modeled, and solid points indicate a significant difference from the first non-zero dose (Supplementary Table 1). Each cell line of each drug was modeled independently.

Pancreatic cancer cells were exposed to various concentrations of the individual drugs to determine the differences in their drug sensitivity and the concentrations appropriate for the drug combination experiments. Cellular proliferation is summarized in Figure 1, and differences among all non-0 doses summarized by cell line and drug in Supplementary Table 1. All cell lines showed significantly reduced proliferation at the highest doses when compared with the lowest non-zero dose. The Capan-1 cell line, which has a *BRCA2* mutation [23], showed a trend towards greater resistance to panobinostat compared with the BxPC-3 and PL45 cells (Figure 1A); the three cell lines showed similar sensitivity to vorinostat (Figure 1B). The PL45 cell line, which is wild-type for *BRCA1* and *BRCA2*, showed a trend towards more resistance to talazoparib and olaparib (PARPis) compared with the BxPC-3 and Capan-1 cell lines (Figures 1C, 1D). The three cell lines showed similar sensitivity to decitabine (Figure 1E). The differences in drug sensitivities of the three pancreatic cell lines are summarized in Figure 1F.

### Synergistic cytotoxicity of HDACis, PARPis, and decitabine

**Figure 2:**
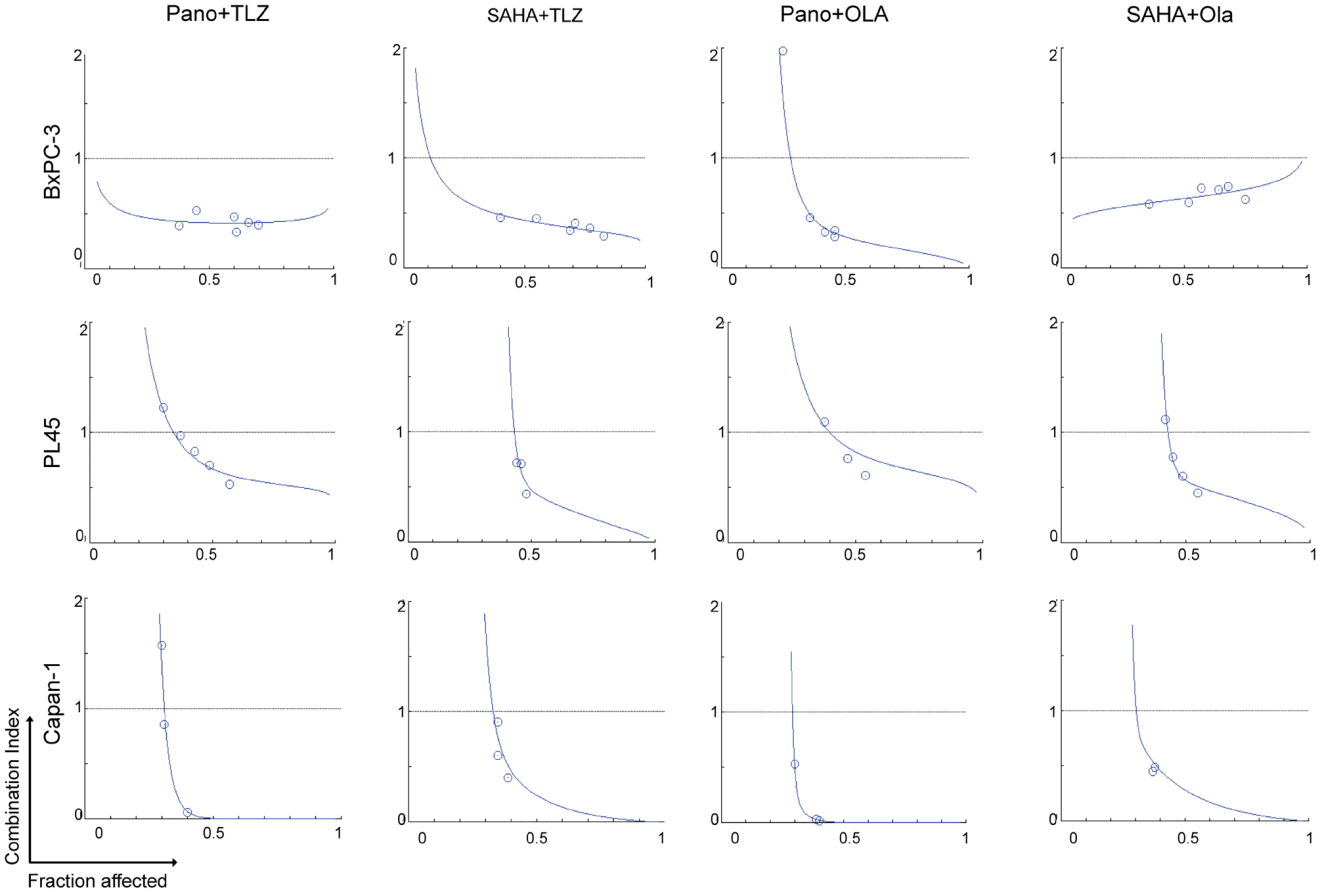
Synergistic cytotoxicity of HDACi and PARPi. Cells were seeded in 96-well plates overnight and exposed to different concentrations of individual drugs or to the two-drug combinations at a constant concentration ratio, and cell proliferation was analyzed after 3 days. The relationships between the calculated combination indexes (CI, Y-axis) and fractions affected (Fa, X-axis) are shown. CI <1.0 indicates synergism. The graphs are representative of two independent experiments. Abbreviations: Ola: Olaparib; Pano: panobinostat; SAHA: vorinostat; TLZ: talazoparib.

HDACis and PARPis are known to have synergistic interactions in hematologic, thyroid, and breast cancer cells [9, 10, 13]. We, therefore, investigated whether these drugs would provide synergistic cytotoxicity in pancreatic cancer cell lines. Cells were exposed to various concentrations of single agents or combinations of two drugs (HDACi + PARPi), using a constant ratio, followed by the MTT assay. Figure 2 shows the calculated CI at increasing drug effects. Significant synergism was noted between HDACis and PARPis, as evidenced by CI values <1 at fraction affected >0.5 in all cell lines.

**Figure 3:**
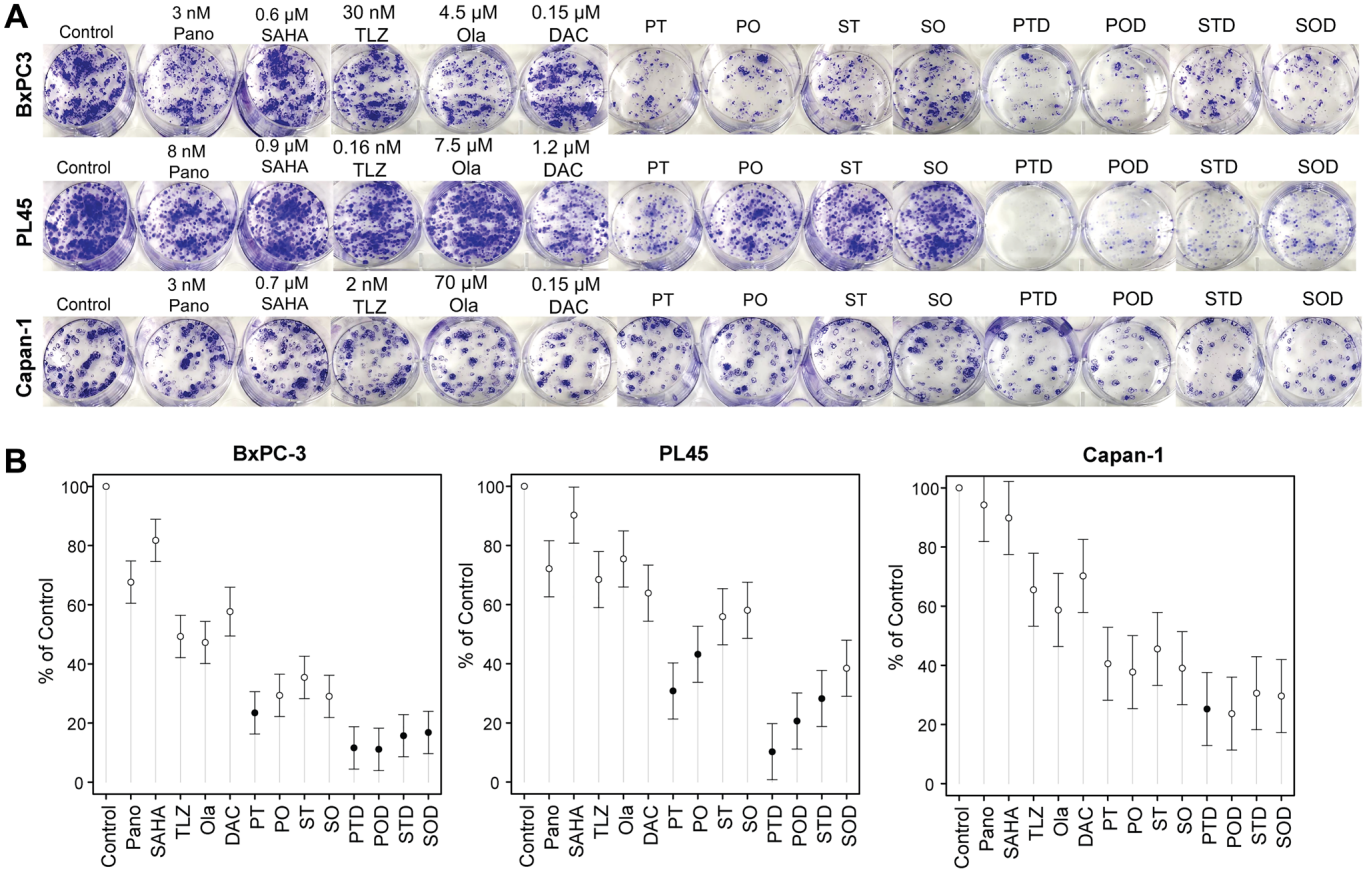
Colony formation assay. Cells were seeded in 6-well plates overnight and exposed to individual drug or drug-combinations for 1–2 weeks and stained as described in the Materials and Methods (**A**). Colony formation is presented relative to control (**B**). Model-adjusted means are shown with 95% confidence intervals, and solid points indicate a significant synergistic difference from all the individual drugs (see Supplementary Table 3). Each cell line of each drug was modeled independently. Abbreviations: DAC/D: decitabine; Ola/O: olaparib; Pano/P: panobinostat; SAHA: vorinostat; TLZ/T: talazoparib.

To further determine the synergistic cytotoxicity of HDACi and PARPi in the three pancreatic cell lines, a clonogenic assay was performed. Quantitative analysis of the colony formation (Figure 3A) showed that exposure of BxPC-3 cells to panobinostat + talazoparib, panobinostat + olaparib, vorinostat + talazoparib, and vorinostat + olaparib resulted in ~77%, ~71%, ~65%, and ~71% inhibition of colony formation, respectively (Figure 3B, Table 1). Panobinostat + talazoparib significantly inhibited colony formation compared with either panobinostat (*P* < 0.0001) or talazoparib (*P* = 0.0008) alone. Similarly, vorinostat + olaparib significantly inhibited colony formation compared with vorinostat (*P* < 0.0001). The inhibition of colony formation mediated by vorinostat + talazoparib was not significantly different than that with the individual drugs. The inhibition of colony formation mediated by panobinostat + olaparib was significantly lower than that noted with panobinostat alone (*p* < 0.0001). The addition of decitabine to each of the two-drug combinations significantly augmented inhibition of colony formation (~83% - 90% inhibition; *P* values < 0.0001), suggesting synergistic cytotoxicity caused by the three-drug combinations to the BxPC-3 cells. Exposure of PL45 cells to panobinostat + talazoparib, panobinostat + olaparib, vorinostat + talazoparib, and vorinostat + olaparib resulted in ~69%, ~57%, ~44%, and ~42% inhibition of colony formation, respectively (Figure 3B, Table 1, Supplementary Table 2). The addition of decitabine to each of these two-drug combinations significantly increased the inhibition rates to ~90%, ~79%, ~72%, and ~62%, respectively (Figure 3B, Table 1). Similar results were obtained when Capan-1 cells were exposed to the two-drug (~54–62% inhibition) or three-drug (~69–76% inhibition) combinations (Figure 3B, Table 1). The inhibition rates of colony formation mediated by all three-drug combinations in the three pancreatic cell lines were mostly statistically higher than those for the individual drugs (Figure 3B, Table 1). These results suggest drug synergistic cytotoxicity using HDACi + PARPi and HDACi + PARPi + decitabine combinations.

**Table 1:** Colony formation and drug-mediated inhibition of cell proliferation in pancreatic cancer cell lines

	Colony formation															
	*BxPC-3*					*PL45*					*Capan-1*				
	Pano	SAHA	TLZ	Ola	DAC	Pano	SAHA	TLZ	Ola	DAC	Pano	SAHA	TLZ	Ola	DAC
Pano+TLZ	<0.0001		<0.0001			<0.0001		<0.0001			<0.0001		0.1235		
Pano+Ola	<0.0001			0.0164		0.0015			0.0003		<0.0001			0.3544	
SAHA+TLZ		<0.0001	0.1630				<0.0001	0.7696				0.0001	0.4327		
SAHA+Ola		<0.0001		0.0135			0.0003		0.2393			<0.0001		0.4640	
Pano+TLZ+DAC	<0.0001		<0.0001		<0.0001	<0.0001		<0.0001		<0.0001	<0.0001		0.0005		<0.0001
Pano+Ola+DAC	<0.0001			<0.0001	<0.0001	<0.0001			<0.0001	<0.0001	<0.0001			0.0041	0.0007
SAHA+TLZ+DAC		<0.0001	<0.0001		<0.0001		<0.0001	<0.0001		<0.0001		<0.0001	0.0041		0.0005
SAHA+Ola+DAC		<0.0001		<0.0001	<0.0001		<0.0001		<0.0001	0.0086		<0.0001		0.0346	<0.0001
Drug-mediated inhibition of cell proliferation^*^															
Pano+TLZ+DAC	<0.0001		<0.0001		<0.0001	<0.0001		<0.0001		<0.0001	<0.0001		0.0043		<0.0001
Pano+Ola+DAC	<0.0001			<0.0001	<0.0001	<0.0001			<0.0001	<0.0001	<0.0001			0.0017	<0.0001
SAHA+TLZ+DAC		<0.0001	0.0020		<0.0001		<0.0001	<0.0001		<0.0001		<0.0001	0.0007		<0.0001
SAHA+Ola+DAC		<0.0001		0.0083	<0.0001		<0.0001		<0.0001	<0.0001		<0.0001		0.0003	<0.0001

**Figure 4:**
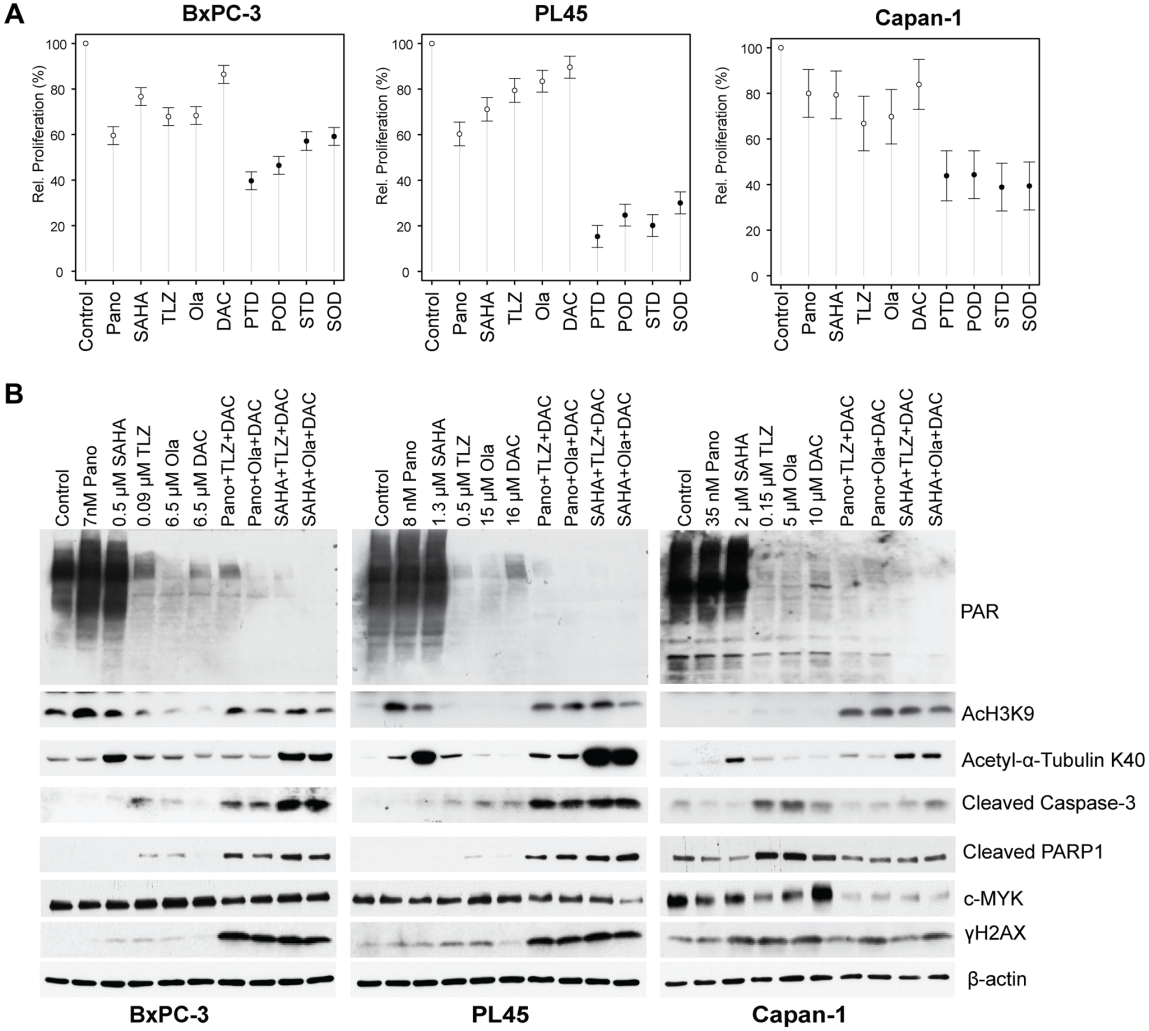
Drug-mediated inhibition of cell proliferation and PARylation, and effects on survival and apoptosis protein markers. Cells were seeded in T25 flasks overnight and exposed to individual drugs or drug combinations for 3 days, harvested, and analyzed for cell proliferation by MTT assay (**A**) and Western blotting (**B**). Model-adjusted means are shown with 95% confidence intervals, and solid points indicate a significant synergistic difference from all the individual drugs (Supplementary Table 2). Each cell line of each drug was modeled independently. Abbreviations: Casp: caspase; DAC/D: decitabine; Ola/O: olaparib; Pano/P: panobinostat; SAHA: vorinostat; TLZ/T: talazoparib.

Comparison of the effects of each drug combination with the effect of the individual drugs ^*^(Table shows the *p*-values for each comparison.) Abbreviations: DAC: decitabine; ND: not determined; Ola: olaparib; Pano: panobinostat; SAHA: vorinostat; TLZ: talazoparib. *P* ≤ 0.05 is considered statistically significant. ^*^Proliferation rates relative to individual drug combinations are displayed in Figures 1, 3 and 4.

The results of the clonogenic assay were consistent with those of the MTT assay for cell proliferation. The addition of the hypomethylating agent decitabine to panobinostat + talazoparib resulted in ~60%, ~85%, and ~57% inhibition of cell proliferation in the BxPC-3, PL45, and Capan-1 cells, respectively; the addition of decitabine to panobinostat + olaparib resulted in ~54%, ~75%, and ~56% inhibition in BxPC-3, PL45, and Capan-1 cells, respectively (Figure 4A). Similar results were obtained when decitabine was combined with vorinostat + talazoparib, which caused ~43%, ~80%, and ~61% inhibition of proliferation in the BxPC-3, PL45, and Capan-1 cells, respectively; the addition of decitabine to vorinostat + olaparib resulted in ~41%, ~70%, and ~61% inhibition of cell proliferation in the BxPC-3, PL45, and Capan-1 cells, respectively (Figure 4A). The three-drug combinations were associated with statistically higher inhibition of proliferation compared to each drug alone (Table 1, Supplementary Table 3).

### The three-drug combinations inhibit PARylation and enhance cleavage of caspase 3 and PARP1

Talazoparib and olaparib are potent inhibitors of PARP. We, therefore, sought to determine whether they also inhibit protein PARylation. While the HDACis panobinostat and vorinostat did not inhibit PARylation, talazoparib and olaparib decreased the PARylation levels in BxPC-3, PL45, and Capan-1 cells, and the addition of decitabine enhanced their inhibitory effects (Figure 4B). Surprisingly, decitabine alone also decreased the levels of PARylation in the three cell lines.

To investigate whether the decrease in colony formation (Figure 3A) and cell proliferation (Figure 4A) were associated with apoptosis, we analyzed the cleavage of caspase 3 and PARP1 (indicators of apoptosis). Figure 4B shows that the triple-drug combinations markedly enhanced both caspase 3 and PARP1 cleavage.

Cells exposed to the triple-drug combinations exhibited increased phosphorylation of histone 2AX. This finding indicates DNA damage response (double strand break formation and/or activation), likely attributed to activation of nuclear DNases, mediated by caspases [24]. These observations are consistent with a decreased level of pro-survival c-MYC in cells exposed to HDACI + PARPi + decitabine (Figure 4B).

### HDACi, PARPi, and decitabine combinations affect the levels of proteins involved in DNA damage response and repair

**Figure 5:**
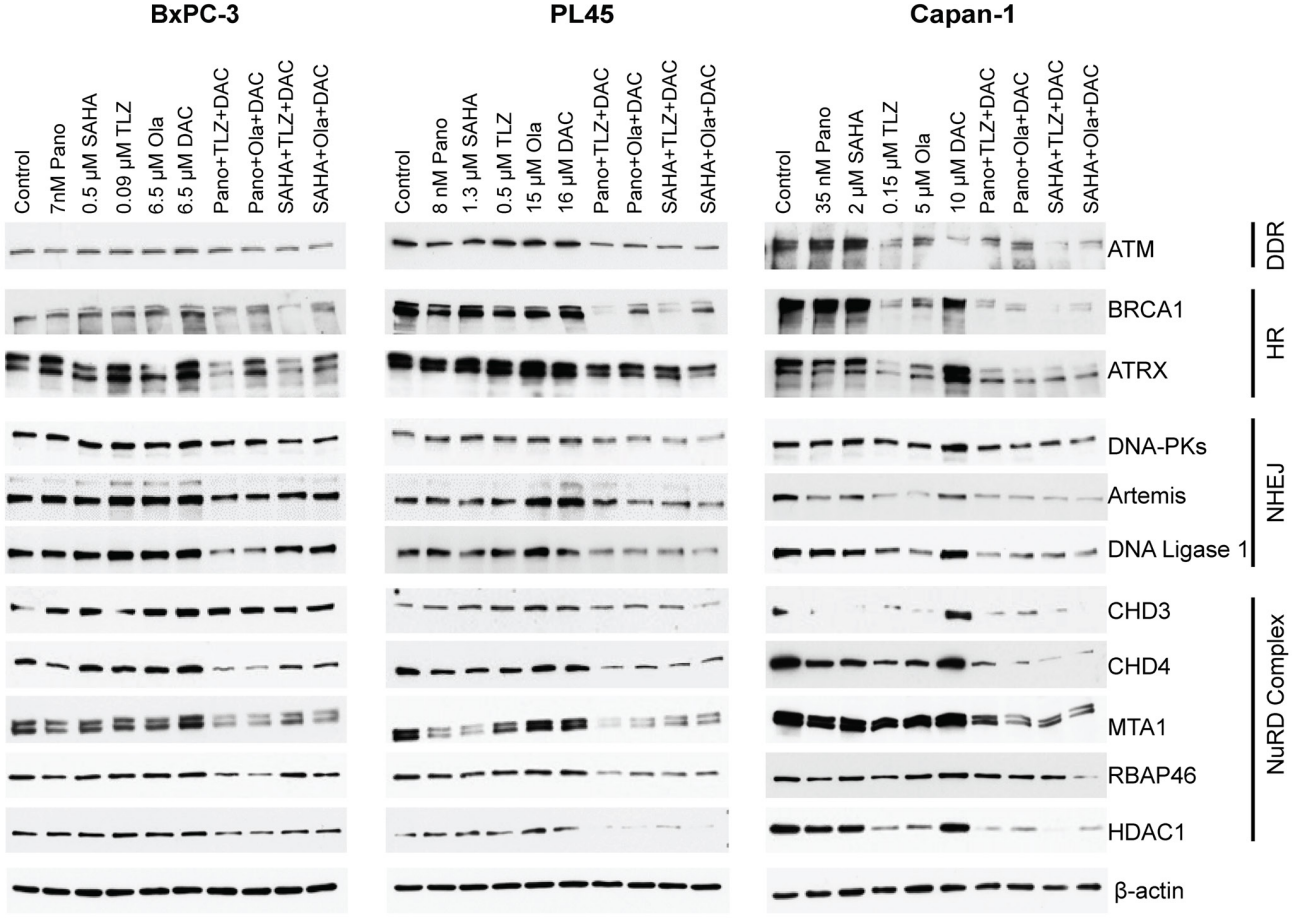
Effects of drugs on the levels of various proteins involved in DNA repair/DNA damage response. Cells were exposed to the indicated drug concentrations for 3 days prior to analysis by Western blotting. Abbreviations: NuRD: nucleosome remodeling and deacetylase; DAC: decitabine; DDR: DNA damage response; HR: Homologous recombination; NHEJ: Non Homologous End Joining; NuRD: The Nucleosome Remodeling and Deacetylase (NuRD); Ola: olaparib; Pano: panobinostat; SAHA: vorinostat; TLZ: talazoparib.

Post-translational modifications (acetylation and PARylation) of proteins associated with DNA repair may affect their stability, as previously described for BRCA1 and UHFR1 [8, 11, 12]. The current study was conducted to assess the effect of HDACi, PARPi, and decitabine, as single agents or in combination, on the levels of the proteins associated with DNA damage response in the aforementioned pancreatic cancer cell lines. The findings indicated that when the cells were exposed to the three-drug combinations, there was a decrease in the levels of the ATM (ataxia-telangiectasia mutated) protein, that is responsible for DNA double-strand break repair and cell cycle checkpoint activation. Additionally, there was also a decrease in the level of BRCA1, which is involved in homologous recombination repair and in the level of the ATRX, a chromatin remodeling protein that participates in homologous recombination repair (Figure 5). In cells exposed to the three-drug combinations, a decrease in the non-homologous end joining repair proteins DNAPKcs, Artemis, and DNA ligase 1 levels was also noted. The cellular DNA damage response is regulated by the nucleosome remodeling and deacetylase (NuRD) complex, which plays a crucial role in chromatin remodeling and deacetylation processes [25] and controls DNA damage signaling and repair [26]. Analysis of some of the subunits of the NuRD complex showed decreased levels of the CHD3, CHD4, MTA1, RBAP46, and HDAC1 proteins in all cell lines exposed to the three-drug combinations (Figure 5). Overall, these results demonstrated that the three-drug combinations decreased the levels of proteins involved in DNA damage response.

## DISCUSSION

This is the first study to demonstrate synergistic interactions between PARPis (olaparib or talazoparib) and panobinostat, vorinostat, and decitabine in pancreatic cancer cell lines with wild-type *BRCA1/2* (BxPC-3 or PL45) or *BRCA2* mutation (Capan-1). An intriguing finding of our study was the observation that the cytotoxicity of PARPis was noted across cell lines and not only in the *BRCA2*-mutated cell line (Capan-1). Synthetic lethality was only expected in Capan-1, but both the BxPC-3 and PL45 cell lines also showed sensitivity to talazoparib and olaparib. The PL45 cell line exhibited weaker sensitivity to these inhibitors compared to the BxPC-3 cell line (Figure 1). This difference in sensitivity to talazoparib and olaparib may be attributed to a non-functional *TP53* mutation in the PL45 cell line, as previously reported [27]. Other investigators have reported similar results in a subset of colorectal cancer cells, which was partly attributed to wild-type *TP53*-mediated suppression of RAD51, a BRCA2-interacting protein [28].

The study demonstrated the following novel findings. The combination of an HDACi (panobinostat or vorinostat) with a PARPi (talazoparib or olaparib) resulted in synergistic cytotoxicity in all pancreatic cell lines tested, as evidenced by a CI of <1.0 for all combinations (Figure 2) and the results of the colony-formation assays (Figure 3A). The addition of the hypomethylating agent decitabine further increased the synergistic cytotoxicity noted with an HDACi (panobinostat or vorinostat) and a PARPi (talazoparib or olaparib) combination (Figure 3B). Several HDACis were investigated, mostly in preclinical studies, for the treatment of pancreatic cancer. M344 (a histone H3 deacetylation inhibitor) has demonstrated antitumor activity as a single agent or combined with gemcitabine *in vitro* and *in vivo* [29]. Trichostatin A has shown suppression of pancreatic adenocarcinoma cell growth, inducing G2 phase cell cycle arrest and apoptotic cell death in cell lines with a mutated p53 gene [30]. Vorinostat has exhibited increased gemcitabine-induced pancreatic cancer cell death through G1 cell cycle arrest [31]. In patients with resected pancreatic ductal cancer, the high expression of HDAC1 activity measured using an HDAC1 inhibitor assay in clinical samples was significantly associated with poor progression-free survival, and distant metastasis-free survival, and indicated that HDAC1 inhibitors may have activity in suppressing metastasis through inhibition of the epithelial-mesenchymal transition (EMT) [32]. CG200745 was shown to overcome resistance of pancreatic cancer cells to gemcitabine [33]. The dual inhibitors Metavert (inhibitor of GSK3B and HDACs) or CUDC-907 (Phosphoinositide 3-kinases/HDAC Inhibitor) and the HDAC inhibitor AES-135 have shown antitumor activity in mouse models of pancreatic cancer [34–36]. Other investigators added vorinostat to capecitabine and radiation therapy in patients with non-metastatic pancreatic cancer and reported that the treatment was well tolerated, and the median overall survival was 1.1 years [37]. Overall, these studies indicate the therapeutic potential of HDAC inhibitors in pancreatic cancer, paving the way for novel therapeutic approaches.

We also explored the mechanisms behind this enhanced cytotoxicity. We observed that the combination treatment caused more cell death by triggering apoptosis, as shown by the increased cleavage of caspase 3 and PARP1 (Figure 4B). The three-drug combination treatment (vorinostat, talazoparib, and decitabine; vorinostat, olaparib, and decitabine; panobinostat, talazoparib, and decitabine; or panobinostat, olaparib, and decitabine) also induced more DNA damage (compared to the individual drugs), as evidenced by the increased phosphorylation of histone 2AX. Furthermore, the combination treatment impaired the DNA repair pathways, as indicated by the decreased levels of ATM, BRCA1, and ATRX proteins (Figure 5). Additionally, the three-drug combination treatment altered the epigenetic regulation of gene expression, as suggested by the reduced levels of NuRD complex subunits. Other investigators have shown that in ovarian cancer cells, the addition of panobinostat to olaparib enhanced the efficacy of olaparib by modulating the expression of genes involved in homologous recombination repair and immune response, and the combination reduced tumor growth and increased tumor cell death, DNA damage, and CD8+ T cell infiltration [38].

Similarly, the combination of vorinostat and talazoparib caused substantial inhibition of pancreatic cancer cell proliferation, especially in PL45 and Capan-1 cells (Figure 3). Cleavage of caspase 3 and PARP1 (Figure 4B), which is indicative of apoptotic cell death, accompanied by the modulation of DNA repair proteins and the downregulation of non-homologous end-joining repair proteins (Figure 5), underscores the disruption of DNA repair processes. The modulation of DNA repair proteins and NuRD complex subunits (including CHD3, CHD4, MTA1, RBAP46, and HDAC1) highlights a comprehensive disruption of DNA repair and chromatin remodeling and epigenetic modifications. The phosphorylation of histone 2AX implies the presence of double-strand DNA breaks, possibly attributed to caspase-mediated activation of nuclear DNases, as previously reported [39]. The downregulation of NuRD complex subunits is consistent with previous reports [40–42], emphasizing the NuRD complex’s role in DNA damage response, DNA repair, and chromatin remodeling.

The combination of vorinostat and olaparib demonstrated synergistic effects against pancreatic cancer cells (Figure 2), with increased apoptotic cell death and DNA damage (Figures 4B and 5). Consistent with previous studies in different cancer types, such as triple-negative breast cancer (TNBC) and ovarian cancer, this finding highlights the potential efficacy of this combination [10, 43]. PTEN loss was suggested as a potential biomarker for predicting response to vorinostat and olaparib in patients with TNBC. Although the exact mechanism is not fully understood, it has been suggested that PTEN expression in TNBC cells can modulate the response to vorinostat and olaparib by affecting the PI3K/AKT/mTOR, HRR, p53, BAX, and autophagy pathways. The interaction of these pathways has synergistic anti-tumor effects in TNBC cells that express functional PTEN [10, 43]. Overall, these findings support the clinical evaluation of vorinostat and olaparib in advanced pancreatic cancer.

The sensitivity of *BRCA*-mutated pancreatic cancer cells to the drug combinations varied. As expected, Capan-1 displayed sensitivity to PARP inhibition, particularly to olaparib. The limited use of HDACis in solid tumors is attributed to pharmacokinetic challenges [44] and resistance mechanisms. In addition, HDACis have been reported to inhibit the functionality of the homologous recombination pathway, leading to a homologous recombination repair-deficient state and, thereby, increasing the effectiveness of PARPis [10]. In addition to olaparib [17], another PARPi, rucaparib, has demonstrated antitumor activity in patients with pancreatic cancer and a known deleterious *BRCA* mutation [45], but antitumor activity has not been noted with veliparib. Furthermore, azacitidine and oxaliplatin combination therapy demonstrated safety with no dose-limiting toxicity in platinum-resistant cancer [46]. Ongoing clinical trials exploring various PARPis aim to further enhance survival outcomes for pancreatic cancer patients.

Although possible mechanisms for the observed synergistic interactions are provided, some limitations of the study must be considered prior to translating these results into clinical trials. As an *in vitro* study, this cell line model cannot predict the complexities of potential interactions within a complex organ, including drug toxicities and pharmacodynamics. A three-dimensional cell model may be more effective in predicting drug effects on tumor tissues. Animal studies may be considered as an alternative, but they have their own inherent drawbacks. The effect of the drugs used in the present study on normal cells should also be considered. Nevertheless, the *in vitro* results presented in this study provide proof of concept for the synergistic interactions of HDACi, PARPi, and decitabine in pancreatic cancer cell lines.

In conclusion, the use of PARPis (olaparib or talazoparib) combined with HDACis (panobinostat, vorinostat), with or without decitabine, demonstrated synergistic cytotoxicity in pancreatic cell lines harboring wild-type or mutated *BRCA1/2* genes. Further research is needed to understand the mechanisms underlying the observed synergistic effects and to identify biomarkers that can predict response to treatment. Collectively, our findings suggest that these combinations should be explored in clinical trials in pancreatic cancer.

## MATERIALS AND METHODS

### Cell lines and drugs

The pancreatic cancer cell lines BxPC-3 (Catalog # CRL-1687), PL45 (CRL-2558), and Capan-1 (HTB-79) were purchased from American Type Culture Collection (ATCC; Manassas, VA, USA). BxPC-3 and PL45 cells possess wild-type *BRCA1* and *BRCA2* genes. Capan-1 cells lack a functional *BRCA2* allele, with single base deletion at *c.6174* resulting in a loss of the C-terminal 1416 amino acids of the proteins [23]. Following ATCC protocols, all cells were cultured in a 5% CO_2_ humidified incubator at 37°C. PL45 and Capan-1 cells were cultured in Dulbecco’s Modified Eagle Medium, while BxPC-3 cells were grown in Roswell Park Memorial Institute 1640 medium. Both media contained 10% heat-inactivated fetal bovine serum along with antibiotics (100 IU/mL penicillin and 100 μg/mL streptomycin).

The HDACis panobinostat and vorinostat, the PARPis talazoparib and olaparib, and the demethylating agent decitabine were obtained from Selleck Chemicals (Houston, TX, USA). Stock solutions were prepared using dimethyl sulfoxide, of which the final concentration did not exceed 0.1% of the total volume.

### Cell proliferation assay and drug synergism

Cell proliferation was determined using the 3-(4,5-dimethylthiazol-2-yl)-2,5-diphenyl tetrazolium bromide (MTT) assay. Briefly, 100 μL of cells (BxPC-3: 3 × 10^4^ cells/mL; PL45: 3.8 × 10^4^ cells/mL; Capan-1: 8 × 10^4^ cells/mL) were seeded per well in a 96-well plate. After 24 hours, the medium was replaced with 100 μL of appropriate medium containing drug(s) aiming for IC_10_ to IC_20_ concentrations and incubated for 3 days. These doses are used to assess synergism between drugs, indicating inhibition of 10% to 20% of cell growth, respectively; a higher drug dose does not allow this assessment. The MTT assay was done by adding 30 μL of MTT (2 mg MTT/mL) in phosphate-buffered saline (PBS) per well and incubating for 4 hours at 37^o^C. The insoluble purple formazan product was dissolved by adding 100 μL of solubilization solution (0.1 N HCl in isopropanol containing 10% Triton X-100) to each well, mixing, and incubating at 37°C overnight. Absorbance at 570 nm was measured using a Victor X3 plate reader (Perkin Elmer Life and Analytical Sciences, Shelton, CT). The rate of cell proliferation was determined relative to the control cells exposed to solvent alone.

To determine drug synergism, cells were seeded in 96-well plates as described above. The medium was changed after 24 hours, and the cells were exposed to various drug combinations aiming for IC_10_ to IC_20_ concentrations for 3 days prior to the MTT assay. Fractions affected (Fa) refer to cell death which was determined using the MTT assay. Drug combination effects were estimated based on the combination index (CI) values calculated using CalcuSyn software (ComboSyn, Inc., Paramus, NJ, USA as previously described [47].

### Colony formation assay

BxPC-3 (1 × 10^3^ cells/mL), PL45 (1.2 × 10^3^ cells/mL), and Capan-1 (3.3 × 10^3^ cells/mL) cells were seeded (3 mL) onto 6-well plates. The next day, the medium was replaced with fresh medium containing drug(s) and incubated at 37°C for 3 days. Then, the medium was replaced with fresh medium without drugs. After 1–2 weeks, formed colonies were fixed using 4% glutaraldehyde for 20 minutes. The colonies were then washed thrice with PBS and stained using 0.5% crystal violet for 15 minutes. Excess stain was removed by washing twice with PBS. The procedures were done at room temperature, and the experiments were repeated at least three times.

### Western blot analysis

Cells (6 mL) were seeded in T25 flasks (BxPC-3: 4.2 × 10^4^ cells; PL45: 5 × 10^4^ cells/mL; Capan-1: 13.3 × 10^4^ cells) overnight. The next day, the old medium was replaced with fresh medium containing drug(s), and the cells were exposed continuously to drug(s) for 3 days. Cells were dissociated from the flask using accutase (MilliporeSigma, St. Louis, MO, USA), harvested, and washed with cold PBS. Cells were lysed with lysis buffer (Cell Signaling Technology, Danvers, MA, USA). Western blot analysis was performed as previously described [13]. Antibodies used for immunoblotting are described in the Supplementary Table 4. The β-actin protein was used as an internal control.

### Statistical analysis

Separately for each drug and for each cell line, mixed effect analysis of variance was used to model the association between cellular proliferation percentage (percentage referenced to the zero-dose sample) and dose (6 discrete levels, excluding dose 0). The same cell culture was used as the basis of all doses of two experiments per day, and experiments were conducted on three separate days. Model-adjusted differences between dose levels were assessed by contrasts with Tukey-adjusted *p*-values using the emmeans package [48], with adjusted means weighted proportionally to covariate marginal frequencies. Mixed effect modeling utilized the nlme package [49, 50]. Cytotoxicity was modeled similarly with relation to drug treatment, separately by cell line, clustering on experiment day, with Hommel-adjusted *p*-values for comparisons between synergistic 3-way drug combinations and component drugs. Colony formation was modeled by analysis of variance with relation to drug treatment, separately by cell line, with Hommel-adjusted *p*-values for comparisons between 2-way and 3-way synergistic drug combinations and component drugs. All statistical modeling of proliferation was conducted using R statistical software (version 4.3.1); a 95% level of statistical confidence was assumed.

## SUPPLEMENTARY MATERIALS





## Abbreviations

ATM: Ataxia-Telangiectasia Mutated protein
ATRX: Alpha Thalassemia/Mental Retardation Syndrome X-Linked protein
BRCA: Breast Cancer gene
CI: Combination Index (used in drug synergism analysis)
c-MYC: Cellular Myelocytomatosis oncogene
DAC: Decitabine
DCIS: Ductal carcinoma *in situ*
DDR: DNA damage response
DNAPKcs: DNA-dependent protein kinase catalytic subunit
Fa: Fractions affected (refers to cell death in drug synergism analysis)
FDA: U.S. Food and Drug Administration
HDACis: histone deacetylase inhibitors
HR: Homologous recombination
IC50: Half-maximal inhibitory concentration
MTT: 3-(4,5-Dimethylthiazol-2-yl)-2,5-Diphenyl Tetrazolium Bromide
NHEJ: Non-Homologous End Joining
NuRD: Nucleosome Remodeling and Deacetylase complex
Ola: Olaparib
Pano: Panobinostat
PARPis: Poly(ADP Ribose) Polymerase Inhibitors
PBS: Phosphate-buffered saline
PD-(L)1: Programmed Death (Ligand) 1
SAHA: Vorinostat
TLZ: Talazoparib
TNF: Tumor Necrosis Factor
UHFR1: Ubiquitin-Specific Histone H2A and H2B Deubiquitinase Factor R1
γ-H2AX: Phosphorylated Histone H2AX
