# Supplementary material for: Synergistic cytotoxicity of histone deacetylase and poly-ADP ribose polymerase inhibitors and decitabine in pancreatic cancer cells: Implications for novel therapy
Source: Oncotarget. 2024 Jun 3;15:361–73. doi: 10.18632/oncotarget.28588 (PMC11146633; doi:10.18632/oncotarget.28588)
Supplement: Supplementary file 1 [file oncotarget-15-28588-s001.pdf]

Synergistic cytotoxicity of histone deacetylase and poly-ADP ribose polymerase inhibitors and decitabine in pancreatic cancer cells: Implications for novel therapy

SUPPLEMENTARY MATERIALS

Supplementary Table 1: Cellular proliferation by drug and cell line: Model-adjusted differences among non-zero doses, with Tukey-adjusted *p*-values. Each cell line of each drug was modeled independently. See Supplementary Table 1

Supplementary Table 2: Colony formation by cell line: Model-adjusted differences among treatments, with Hommel-adjusted *p*-values

Cell line	Contrast	Estimate	SE	CI 95 Min	CI 95 Max	Hommel *P*-value
BxPC-3	PT - Pano	−44.2	5.2	−54.3	−34.1	<.0001
BxPC-3	PT - TLZ	−25.8	5.2	−35.9	−15.7	0.0008
BxPC-3	PO - Pano	−38.3	5.2	−48.4	−28.2	<.0001
BxPC-3	PO - Ola	−17.9	5.2	−28.0	−7.8	0.08
BxPC-3	ST - SAHA	−46.3	5.2	−56.4	−36.2	<.0001
BxPC-3	ST - TLZ	−13.8	5.2	−23.9	−3.7	0.28
BxPC-3	SO - SAHA	−52.7	5.2	−62.8	−42.6	<.0001
BxPC-3	SO - Ola	−18.2	5.2	−28.3	−8.1	0.07
BxPC-3	PTD - Pano	−56.0	5.2	−66.1	−45.9	<.0001
BxPC-3	PTD - TLZ	−37.6	5.2	−47.8	−27.5	<.0001
BxPC-3	PTD - Dec	−46.1	5.6	−57.0	−35.1	<.0001
BxPC-3	POD - Pano	−56.5	5.2	−66.6	−46.4	<.0001
BxPC-3	POD - Ola	−36.1	5.2	−46.2	−26.0	<.0001
BxPC-3	POD - Dec	−46.6	5.6	−57.5	−35.6	<.0001
BxPC-3	STD - SAHA	−66.0	5.2	−76.2	−55.9	<.0001
BxPC-3	STD - TLZ	−33.5	5.2	−43.7	−23.4	<.0001
BxPC-3	STD - Dec	−42.0	5.6	−52.9	−31.0	<.0001
BxPC-3	SOD - SAHA	−64.9	5.2	−75.1	−54.8	<.0001
BxPC-3	SOD - Ola	−30.4	5.2	−40.5	−20.3	<.0001
BxPC-3	SOD - Dec	−40.9	5.6	−51.8	−30.0	<.0001
PL45	PT - Pano	−41.3	6.8	−54.7	−27.9	<.0001
PL45	PT - TLZ	−37.6	6.8	−51.1	−24.2	0.0004
PL45	PO - Pano	−28.9	6.8	−42.3	−15.5	0.019
PL45	PO - Ola	−32.2	6.8	−45.7	−18.8	0.004
PL45	ST - SAHA	−34.4	6.8	−47.8	−20.9	0.002
PL45	ST - TLZ	−12.6	6.8	−26.0	0.8	0.82
PL45	SO - SAHA	−32.2	6.8	−45.6	−18.8	0.005
PL45	SO - Ola	−17.4	6.8	−30.8	−4.0	0.74
PL45	PTD - Pano	−61.9	6.8	−75.3	−48.5	<.0001
PL45	PTD - TLZ	−58.2	6.8	−71.6	−44.8	<.0001

PL45	PTD - Dec	−53.6	6.8	−67.1	−40.2	<.0001
PL45	POD - Pano	−51.5	6.8	−64.9	−38.1	<.0001
PL45	POD - Ola	−54.8	6.8	−68.2	−41.4	<.0001
PL45	POD - Dec	−43.3	6.8	−56.7	−29.8	<.0001
PL45	STD - SAHA	−62.0	6.8	−75.4	−48.6	<.0001
PL45	STD - TLZ	−40.2	6.8	−53.6	−26.8	0.0001
PL45	STD - Dec	−35.7	6.8	−49.1	−22.3	0.001
PL45	SOD - SAHA	−51.8	6.8	−65.2	−38.4	<.0001
PL45	SOD - Ola	−37.0	6.8	−50.4	−23.6	0.0006
PL45	SOD - Dec	−25.4	6.8	−38.8	−12.0	0.069
Capan-1	PT - Pano	−53.7	8.9	−71.1	−36.2	<.0001
Capan-1	PT - TLZ	−25.0	8.9	−42.4	−7.5	0.59
Capan-1	PO - Pano	−56.5	8.9	−74.0	−39.0	<.0001
Capan-1	PO - Ola	−21.0	8.9	−38.4	−3.5	0.59
Capan-1	ST - SAHA	−44.3	8.9	−61.7	−26.8	0.004
Capan-1	ST - TLZ	−20.0	8.9	−37.5	−2.6	0.59
Capan-1	SO - SAHA	−50.8	8.9	−68.2	−33.3	0.0003
Capan-1	SO - Ola	−19.7	8.9	−37.1	−2.2	0.59
Capan-1	PTD - Pano	−69.0	8.9	−86.4	−51.5	<.0001
Capan-1	PTD - TLZ	−40.3	8.9	−57.8	−22.9	0.014
Capan-1	PTD - Dec	−45.0	8.9	−62.4	−27.5	0.003
Capan-1	POD - Pano	−70.6	8.9	−88.0	−53.1	<.0001
Capan-1	POD - Ola	−35.0	8.9	−52.5	−17.6	0.07
Capan-1	POD - Dec	−46.6	8.9	−64.0	−29.1	0.002
Capan-1	STD - SAHA	−59.2	8.9	−76.7	−41.8	<.0001
Capan-1	STD - TLZ	−35.0	8.9	−52.4	−17.5	0.07
Capan-1	STD - Dec	−39.6	8.9	−57.1	−22.2	0.018
Capan-1	SOD - SAHA	−60.2	8.9	−77.7	−42.7	<.0001
Capan-1	SOD - Ola	−29.1	8.9	−46.5	−11.6	0.39
Capan-1	SOD - Dec	−40.6	8.9	−58.0	−23.1	0.012

Each cell line was modeled independently.

Supplementary Table 3: Cytotoxicity by cell line: Model-adjusted differences among treatments, with Hommel-adjusted *p*-values

CellLine	Contrast	Estimate	SE	CI 95 Min	CI 95 Max	Hommel *P*-value
BxPC-3	PTD - Pano	−19.9	2.3	−24.5	−15.3	<.0001
BxPC-3	PTD - TLZ	−28.2	2.3	−32.8	−23.6	<.0001
BxPC-3	PTD - DAC	−46.7	2.3	−51.3	−42.1	<.0001
BxPC-3	POD - Pano	−13.1	2.3	−17.7	−8.5	<.0001
BxPC-3	POD - Ola	−21.9	2.3	−26.4	−17.3	<.0001
BxPC-3	POD - DAC	−40.0	2.3	−44.5	−35.4	<.0001
BxPC-3	STD - SAHA	−19.5	2.4	−24.3	−14.8	<.0001
BxPC-3	STD - TLZ	−10.7	2.4	−15.5	−6.0	0.003
BxPC-3	STD - DAC	−29.3	2.4	−34.1	−24.5	<.0001

BxPC-3	SOD - SAHA	−17.5	2.3	−22.1	−12.9	<.0001
BxPC-3	SOD - Ola	−9.2	2.3	−13.8	−4.6	0.007
BxPC-3	SOD - DAC	−27.3	2.3	−31.9	−22.7	<.0001
PL45	PTD - Pano	−45.0	2.5	−49.8	−40.1	<.0001
PL45	PTD - TLZ	−64.1	2.5	−69.0	−59.2	<.0001
PL45	PTD - DAC	−74.3	2.3	−78.7	−69.8	<.0001
PL45	POD - Pano	−35.6	2.5	−40.5	−30.7	<.0001
PL45	POD - Ola	−58.8	2.3	−63.2	−54.3	<.0001
PL45	POD - DAC	−64.9	2.3	−69.4	−60.5	<.0001
PL45	STD - SAHA	−51.0	2.5	−55.8	−46.1	<.0001
PL45	STD - TLZ	−59.3	2.5	−64.1	−54.4	<.0001
PL45	STD - DAC	−69.4	2.3	−73.9	−65.0	<.0001
PL45	SOD - SAHA	−41.1	2.5	−45.9	−36.2	<.0001
PL45	SOD - Ola	−53.4	2.3	−57.8	−48.9	<.0001
PL45	SOD - DAC	−59.5	2.3	−64.0	−55.1	<.0001
Capan-1	PTD - Pano	−36.1	4.2	−44.4	−27.9	<.0001
Capan-1	PTD - TLZ	−22.9	5.2	−33.1	−12.8	0.007
Capan-1	PTD - DAC	−40.1	4.5	−48.8	−31.3	<.0001
Capan-1	POD - Pano	−35.7	3.9	−43.3	−28.1	<.0001
Capan-1	POD - Ola	−25.4	4.9	−35.0	−15.9	0.002
Capan-1	POD - DAC	−39.6	4.2	−47.8	−31.4	<.0001
Capan-1	STD - SAHA	−40.5	3.9	−48.1	−32.9	<.0001
Capan-1	STD - TLZ	−27.9	4.9	−37.5	−18.4	0.001
Capan-1	STD - DAC	−45.0	4.2	−53.3	−36.8	<.0001
Capan-1	SOD - SAHA	−40.0	3.9	−47.6	−32.4	<.0001
Capan-1	SOD - Ola	−30.4	4.9	−39.9	−20.8	0.0005
Capan-1	SOD - DAC	−44.5	4.2	−52.8	−36.3	<.0001

Each cell line was modeled independently. Abbreviations: DAC/D: decitabine; Ola/O: olaparib; Pano/P: panobinostat; SAHA: vorinostat; TLZ/T: talazoparib.

Supplementary Table 4: Antibodies used in Western blotting analyses, their respective antigen targets, and sourcing information

Antigen	Company/Cat. No.	Source*	Dilution**
β-ACTIN	Sigma/A5316	Mouse	6000
Ac H3 K9	Active Motif/39917	Rabbit	2000
Ac α-Tubulin K40	Cell Signaling/5335	Rabbit	3000
Artemis	Cell Signaling/13381	Rabbit	3000
ATM	Cell Signaling/2873	Rabbit	2500
ATRX	Cell Signaling/10321	Rabbit	3000
BRCA1	Cell Signaling/14823	Rabbit	2000
CHD3	Cell Signaling/4241	Rabbit	2000
CHD4	Cell Signaling/12011	Rabbit	2000
Cleaved CASPASE 3	Cell Signaling/9661	Rabbit	2500
Cleaved PARP1 (Asp214)	Cell Signaling/5625	Rabbit	2000

DNA Ligase 1	GeneTex/GTX70141	Mouse	2500
DNA-PKcs	Cell Signaling/4602	Rabbit	3000
HDAC1	Cell Signaling/34589	Rabbit	2500
γ-H2AX	Cell Signaling/2577	Rabbit	1500
MTA1	Cell Signaling/5647	Rabbit	2000
c-MYC	Cell Signaling/5605	Rabbit	3000
PAR	Bio-Techne/4335-MC-100	Mouse	2000
RBAP46	Cell Signaling/9067	Rabbit	3000

*Anti-rabbit IgG or anti-mouse IgG was used as secondary antibody, obtained from Bio-Rad Lab. **Working solution, fold dilution in PBS with 0.1% Tween 20.
