# Supplementary material for: Synergistic cytotoxicity of histone deacetylase and poly-ADP ribose polymerase inhibitors and decitabine in pancreatic cancer cells: Implications for novel therapy
Source: Oncotarget. 2024 Jun 3;15:361–73. doi: 10.18632/oncotarget.28588 (PMC11146633; doi:10.18632/oncotarget.28588)
Supplement: Supplementary file 2 [file oncotarget-15-28588-s002.docx]

**Supplemental Table 1: Cellular proliferation by drug and cell line: Model-adjusted differences among non-zero doses, with Tukey-adjusted *p*‑values. Each cell line of each drug was modeled independently.**

| Drug | Cell Line | Contrast | Estimate | SE | CI95Min | CI95Max | TukeyPValue |
| --- | --- | --- | --- | --- | --- | --- | --- |
| Panobinostat | BxPC-3 | Dose10 - Dose5 | -20.6 | 2.8 | -26.1 | -15.2 | <.0001 |
| Panobinostat | BxPC-3 | Dose15 - Dose5 | -33.0 | 2.8 | -38.4 | -27.6 | <.0001 |
| Panobinostat | BxPC-3 | Dose15 - Dose10 | -12.4 | 2.8 | -17.8 | -6.9 | 0.001 |
| Panobinostat | BxPC-3 | Dose20 - Dose5 | -41.0 | 2.8 | -46.4 | -35.6 | <.0001 |
| Panobinostat | BxPC-3 | Dose20 - Dose10 | -20.4 | 2.8 | -25.8 | -15.0 | <.0001 |
| Panobinostat | BxPC-3 | Dose20 - Dose15 | -8.0 | 2.8 | -13.4 | -2.6 | 0.07 |
| Panobinostat | BxPC-3 | Dose25 - Dose5 | -49.3 | 2.8 | -54.7 | -43.8 | <.0001 |
| Panobinostat | BxPC-3 | Dose25 - Dose10 | -28.6 | 2.8 | -34.0 | -23.2 | <.0001 |
| Panobinostat | BxPC-3 | Dose25 - Dose15 | -16.3 | 2.8 | -21.7 | -10.8 | <.0001 |
| Panobinostat | BxPC-3 | Dose25 - Dose20 | -8.2 | 2.8 | -13.7 | -2.8 | 0.059 |
| Panobinostat | BxPC-3 | Dose30 - Dose5 | -57.3 | 2.8 | -62.7 | -51.9 | <.0001 |
| Panobinostat | BxPC-3 | Dose30 - Dose10 | -36.6 | 2.8 | -42.1 | -31.2 | <.0001 |
| Panobinostat | BxPC-3 | Dose30 - Dose15 | -24.3 | 2.8 | -29.7 | -18.9 | <.0001 |
| Panobinostat | BxPC-3 | Dose30 - Dose20 | -16.3 | 2.8 | -21.7 | -10.9 | <.0001 |
| Panobinostat | BxPC-3 | Dose30 - Dose25 | -8.0 | 2.8 | -13.5 | -2.6 | 0.069 |
| Panobinostat | PL45 | Dose10 - Dose5 | -22.5 | 1.9 | -26.2 | -18.7 | <.0001 |
| Panobinostat | PL45 | Dose15 - Dose5 | -48.8 | 1.9 | -52.6 | -45.0 | <.0001 |
| Panobinostat | PL45 | Dose15 - Dose10 | -26.3 | 1.9 | -30.1 | -22.5 | <.0001 |
| Panobinostat | PL45 | Dose20 - Dose5 | -65.6 | 1.9 | -69.4 | -61.8 | <.0001 |
| Panobinostat | PL45 | Dose20 - Dose10 | -43.1 | 1.9 | -46.9 | -39.3 | <.0001 |
| Panobinostat | PL45 | Dose20 - Dose15 | -16.8 | 1.9 | -20.6 | -13.0 | <.0001 |
| Panobinostat | PL45 | Dose25 - Dose5 | -77.2 | 1.9 | -80.9 | -73.4 | <.0001 |
| Panobinostat | PL45 | Dose25 - Dose10 | -54.7 | 1.9 | -58.5 | -50.9 | <.0001 |
| Panobinostat | PL45 | Dose25 - Dose15 | -28.4 | 1.9 | -32.1 | -24.6 | <.0001 |
| Panobinostat | PL45 | Dose25 - Dose20 | -11.6 | 1.9 | -15.4 | -7.8 | <.0001 |
| Panobinostat | PL45 | Dose30 - Dose5 | -86.2 | 1.9 | -90.0 | -82.4 | <.0001 |
| Panobinostat | PL45 | Dose30 - Dose10 | -63.7 | 1.9 | -67.5 | -59.9 | <.0001 |
| Panobinostat | PL45 | Dose30 - Dose15 | -37.4 | 1.9 | -41.2 | -33.6 | <.0001 |
| Panobinostat | PL45 | Dose30 - Dose20 | -20.6 | 1.9 | -24.4 | -16.8 | <.0001 |
| Panobinostat | PL45 | Dose30 - Dose25 | -9.0 | 1.9 | -12.8 | -5.2 | 0.0009 |
| Panobinostat | Capan1 | Dose10 - Dose5 | -5.4 | 3.3 | -11.8 | 1.1 | 0.58 |
| Panobinostat | Capan1 | Dose15 - Dose5 | -10.2 | 3.3 | -16.6 | -3.7 | 0.048 |
| Panobinostat | Capan1 | Dose15 - Dose10 | -4.8 | 3.4 | -11.5 | 1.9 | 0.73 |
| Panobinostat | Capan1 | Dose20 - Dose5 | -14.7 | 3.1 | -20.8 | -8.5 | 0.001 |
| Panobinostat | Capan1 | Dose20 - Dose10 | -9.3 | 3.3 | -15.7 | -2.8 | 0.09 |
| Panobinostat | Capan1 | Dose20 - Dose15 | -4.5 | 3.3 | -10.9 | 2.0 | 0.75 |
| Panobinostat | Capan1 | Dose25 - Dose5 | -24.7 | 3.1 | -30.8 | -18.6 | <.0001 |
| Panobinostat | Capan1 | Dose25 - Dose10 | -19.3 | 3.3 | -25.8 | -12.9 | <.0001 |
| Panobinostat | Capan1 | Dose25 - Dose15 | -14.5 | 3.3 | -21.0 | -8.1 | 0.002 |
| Panobinostat | Capan1 | Dose25 - Dose20 | -10.0 | 3.1 | -16.2 | -3.9 | 0.037 |
| Panobinostat | Capan1 | Dose30 - Dose5 | -38.6 | 3.1 | -44.7 | -32.5 | <.0001 |
| Panobinostat | Capan1 | Dose30 - Dose10 | -33.2 | 3.3 | -39.7 | -26.8 | <.0001 |
| Panobinostat | Capan1 | Dose30 - Dose15 | -28.4 | 3.3 | -34.9 | -22.0 | <.0001 |
| Panobinostat | Capan1 | Dose30 - Dose20 | -23.9 | 3.1 | -30.0 | -17.8 | <.0001 |
| Panobinostat | Capan1 | Dose30 - Dose25 | -13.9 | 3.1 | -20.0 | -7.7 | 0.002 |
| Vorinostat_SAHA | BxPC-3 | Dose2 - Dose1 | -18.4 | 3.4 | -25.0 | -11.7 | 0.0001 |
| Vorinostat_SAHA | BxPC-3 | Dose3 - Dose1 | -39.0 | 3.4 | -45.7 | -32.4 | <.0001 |
| Vorinostat_SAHA | BxPC-3 | Dose3 - Dose2 | -20.6 | 3.4 | -27.3 | -14.0 | <.0001 |
| Vorinostat_SAHA | BxPC-3 | Dose4 - Dose1 | -50.6 | 3.4 | -57.2 | -43.9 | <.0001 |
| Vorinostat_SAHA | BxPC-3 | Dose4 - Dose2 | -32.2 | 3.4 | -38.8 | -25.5 | <.0001 |
| Vorinostat_SAHA | BxPC-3 | Dose4 - Dose3 | -11.5 | 3.4 | -18.2 | -4.9 | 0.022 |
| Vorinostat_SAHA | BxPC-3 | Dose5 - Dose1 | -57.3 | 3.4 | -63.9 | -50.6 | <.0001 |
| Vorinostat_SAHA | BxPC-3 | Dose5 - Dose2 | -38.9 | 3.4 | -45.5 | -32.3 | <.0001 |
| Vorinostat_SAHA | BxPC-3 | Dose5 - Dose3 | -18.3 | 3.4 | -24.9 | -11.6 | 0.0001 |
| Vorinostat_SAHA | BxPC-3 | Dose5 - Dose4 | -6.7 | 3.4 | -13.4 | -0.1 | 0.38 |
| Vorinostat_SAHA | BxPC-3 | Dose6 - Dose1 | -63.4 | 3.4 | -70.1 | -56.8 | <.0001 |
| Vorinostat_SAHA | BxPC-3 | Dose6 - Dose2 | -45.0 | 3.4 | -51.7 | -38.4 | <.0001 |
| Vorinostat_SAHA | BxPC-3 | Dose6 - Dose3 | -24.4 | 3.4 | -31.0 | -17.7 | <.0001 |
| Vorinostat_SAHA | BxPC-3 | Dose6 - Dose4 | -12.9 | 3.4 | -19.5 | -6.2 | 0.009 |
| Vorinostat_SAHA | BxPC-3 | Dose6 - Dose5 | -6.1 | 3.4 | -12.8 | 0.5 | 0.48 |
| Vorinostat_SAHA | PL45 | Dose2 - Dose1 | -15.0 | 3.0 | -20.9 | -9.2 | 0.0004 |
| Vorinostat_SAHA | PL45 | Dose3 - Dose1 | -54.7 | 3.0 | -60.5 | -48.8 | <.0001 |
| Vorinostat_SAHA | PL45 | Dose3 - Dose2 | -39.7 | 2.7 | -44.9 | -34.5 | <.0001 |
| Vorinostat_SAHA | PL45 | Dose4 - Dose1 | -77.5 | 3.0 | -83.4 | -71.7 | <.0001 |
| Vorinostat_SAHA | PL45 | Dose4 - Dose2 | -62.5 | 2.7 | -67.7 | -57.3 | <.0001 |
| Vorinostat_SAHA | PL45 | Dose4 - Dose3 | -22.8 | 2.7 | -28.0 | -17.6 | <.0001 |
| Vorinostat_SAHA | PL45 | Dose5 - Dose1 | -85.2 | 3.0 | -91.1 | -79.4 | <.0001 |
| Vorinostat_SAHA | PL45 | Dose5 - Dose2 | -70.2 | 2.7 | -75.4 | -65.0 | <.0001 |
| Vorinostat_SAHA | PL45 | Dose5 - Dose3 | -30.6 | 2.7 | -35.8 | -25.4 | <.0001 |
| Vorinostat_SAHA | PL45 | Dose5 - Dose4 | -7.7 | 2.7 | -12.9 | -2.5 | 0.07 |
| Vorinostat_SAHA | PL45 | Dose6 - Dose1 | -88.9 | 3.0 | -94.8 | -83.1 | <.0001 |
| Vorinostat_SAHA | PL45 | Dose6 - Dose2 | -73.9 | 2.7 | -79.1 | -68.7 | <.0001 |
| Vorinostat_SAHA | PL45 | Dose6 - Dose3 | -34.3 | 2.7 | -39.5 | -29.1 | <.0001 |
| Vorinostat_SAHA | PL45 | Dose6 - Dose4 | -11.4 | 2.7 | -16.6 | -6.2 | 0.003 |
| Vorinostat_SAHA | PL45 | Dose6 - Dose5 | -3.7 | 2.7 | -8.9 | 1.5 | 0.73 |
| Vorinostat_SAHA | Capan1 | Dose2 - Dose1 | -11.7 | 2.6 | -16.8 | -6.6 | 0.001 |
| Vorinostat_SAHA | Capan1 | Dose3 - Dose1 | -27.3 | 2.6 | -32.3 | -22.2 | <.0001 |
| Vorinostat_SAHA | Capan1 | Dose3 - Dose2 | -15.6 | 2.6 | -20.6 | -10.5 | <.0001 |
| Vorinostat_SAHA | Capan1 | Dose4 - Dose1 | -42.0 | 2.6 | -47.1 | -36.9 | <.0001 |
| Vorinostat_SAHA | Capan1 | Dose4 - Dose2 | -30.3 | 2.6 | -35.4 | -25.2 | <.0001 |
| Vorinostat_SAHA | Capan1 | Dose4 - Dose3 | -14.8 | 2.6 | -19.8 | -9.7 | <.0001 |
| Vorinostat_SAHA | Capan1 | Dose5 - Dose1 | -54.3 | 2.6 | -59.4 | -49.2 | <.0001 |
| Vorinostat_SAHA | Capan1 | Dose5 - Dose2 | -42.6 | 2.6 | -47.7 | -37.5 | <.0001 |
| Vorinostat_SAHA | Capan1 | Dose5 - Dose3 | -27.0 | 2.6 | -32.1 | -22.0 | <.0001 |
| Vorinostat_SAHA | Capan1 | Dose5 - Dose4 | -12.3 | 2.6 | -17.4 | -7.2 | 0.0007 |
| Vorinostat_SAHA | Capan1 | Dose6 - Dose1 | -65.4 | 2.6 | -70.5 | -60.3 | <.0001 |
| Vorinostat_SAHA | Capan1 | Dose6 - Dose2 | -53.7 | 2.6 | -58.8 | -48.6 | <.0001 |
| Vorinostat_SAHA | Capan1 | Dose6 - Dose3 | -38.2 | 2.6 | -43.2 | -33.1 | <.0001 |
| Vorinostat_SAHA | Capan1 | Dose6 - Dose4 | -23.4 | 2.6 | -28.5 | -18.3 | <.0001 |
| Vorinostat_SAHA | Capan1 | Dose6 - Dose5 | -11.1 | 2.6 | -16.2 | -6.1 | 0.002 |
| Talazoparib | BxPC-3 | Dose0.5 - Dose0.25 | -6.1 | 1.4 | -8.8 | -3.4 | 0.002 |
| Talazoparib | BxPC-3 | Dose0.75 - Dose0.25 | -11.6 | 1.4 | -14.3 | -8.9 | <.0001 |
| Talazoparib | BxPC-3 | Dose0.75 - Dose0.5 | -5.5 | 1.4 | -8.2 | -2.8 | 0.005 |
| Talazoparib | BxPC-3 | Dose1 - Dose0.25 | -16.1 | 1.4 | -18.8 | -13.4 | <.0001 |
| Talazoparib | BxPC-3 | Dose1 - Dose0.5 | -10.0 | 1.4 | -12.7 | -7.3 | <.0001 |
| Talazoparib | BxPC-3 | Dose1 - Dose0.75 | -4.5 | 1.4 | -7.2 | -1.8 | 0.03 |
| Talazoparib | BxPC-3 | Dose1.25 - Dose0.25 | -18.8 | 1.4 | -21.5 | -16.1 | <.0001 |
| Talazoparib | BxPC-3 | Dose1.25 - Dose0.5 | -12.7 | 1.4 | -15.4 | -10.0 | <.0001 |
| Talazoparib | BxPC-3 | Dose1.25 - Dose0.75 | -7.2 | 1.4 | -9.9 | -4.5 | 0.0002 |
| Talazoparib | BxPC-3 | Dose1.25 - Dose1 | -2.7 | 1.4 | -5.4 | 0.0 | 0.4 |
| Talazoparib | BxPC-3 | Dose1.5 - Dose0.25 | -27.1 | 1.4 | -29.8 | -24.4 | <.0001 |
| Talazoparib | BxPC-3 | Dose1.5 - Dose0.5 | -21.0 | 1.4 | -23.7 | -18.3 | <.0001 |
| Talazoparib | BxPC-3 | Dose1.5 - Dose0.75 | -15.5 | 1.4 | -18.2 | -12.8 | <.0001 |
| Talazoparib | BxPC-3 | Dose1.5 - Dose1 | -11.0 | 1.4 | -13.7 | -8.3 | <.0001 |
| Talazoparib | BxPC-3 | Dose1.5 - Dose1.25 | -8.3 | 1.4 | -11.0 | -5.6 | <.0001 |
| Talazoparib | PL45 | Dose0.5 - Dose0.25 | -1.9 | 1.3 | -4.4 | 0.6 | 0.68 |
| Talazoparib | PL45 | Dose0.75 - Dose0.25 | -3.5 | 1.2 | -5.9 | -1.2 | 0.067 |
| Talazoparib | PL45 | Dose0.75 - Dose0.5 | -1.7 | 1.3 | -4.1 | 0.8 | 0.78 |
| Talazoparib | PL45 | Dose1 - Dose0.25 | -6.4 | 1.2 | -8.8 | -4.1 | 0.0002 |
| Talazoparib | PL45 | Dose1 - Dose0.5 | -4.5 | 1.3 | -7.0 | -2.1 | 0.015 |
| Talazoparib | PL45 | Dose1 - Dose0.75 | -2.9 | 1.2 | -5.3 | -0.5 | 0.19 |
| Talazoparib | PL45 | Dose1.25 - Dose0.25 | -10.4 | 1.2 | -12.8 | -8.1 | <.0001 |
| Talazoparib | PL45 | Dose1.25 - Dose0.5 | -8.5 | 1.3 | -11.0 | -6.1 | <.0001 |
| Talazoparib | PL45 | Dose1.25 - Dose0.75 | -6.9 | 1.2 | -9.2 | -4.5 | <.0001 |
| Talazoparib | PL45 | Dose1.25 - Dose1 | -4.0 | 1.2 | -6.4 | -1.6 | 0.028 |
| Talazoparib | PL45 | Dose1.5 - Dose0.25 | -24.5 | 1.2 | -26.9 | -22.2 | <.0001 |
| Talazoparib | PL45 | Dose1.5 - Dose0.5 | -22.6 | 1.3 | -25.1 | -20.2 | <.0001 |
| Talazoparib | PL45 | Dose1.5 - Dose0.75 | -21.0 | 1.2 | -23.3 | -18.6 | <.0001 |
| Talazoparib | PL45 | Dose1.5 - Dose1 | -18.1 | 1.2 | -20.5 | -15.7 | <.0001 |
| Talazoparib | PL45 | Dose1.5 - Dose1.25 | -14.1 | 1.2 | -16.5 | -11.7 | <.0001 |
| Talazoparib | Capan1 | Dose0.5 - Dose0.25 | -3.4 | 1.5 | -6.4 | -0.5 | 0.22 |
| Talazoparib | Capan1 | Dose0.75 - Dose0.25 | -6.8 | 1.5 | -9.7 | -3.9 | 0.001 |
| Talazoparib | Capan1 | Dose0.75 - Dose0.5 | -3.4 | 1.5 | -6.3 | -0.5 | 0.24 |
| Talazoparib | Capan1 | Dose1 - Dose0.25 | -7.1 | 1.6 | -10.1 | -4.0 | 0.001 |
| Talazoparib | Capan1 | Dose1 - Dose0.5 | -3.6 | 1.6 | -6.7 | -0.6 | 0.22 |
| Talazoparib | Capan1 | Dose1 - Dose0.75 | -0.3 | 1.6 | -3.3 | 2.8 | 1 |
| Talazoparib | Capan1 | Dose1.25 - Dose0.25 | -9.7 | 1.6 | -12.7 | -6.6 | <.0001 |
| Talazoparib | Capan1 | Dose1.25 - Dose0.5 | -6.2 | 1.6 | -9.3 | -3.2 | 0.006 |
| Talazoparib | Capan1 | Dose1.25 - Dose0.75 | -2.9 | 1.6 | -5.9 | 0.2 | 0.46 |
| Talazoparib | Capan1 | Dose1.25 - Dose1 | -2.6 | 1.6 | -5.8 | 0.6 | 0.6 |
| Talazoparib | Capan1 | Dose1.5 - Dose0.25 | -18.7 | 1.5 | -21.6 | -15.8 | <.0001 |
| Talazoparib | Capan1 | Dose1.5 - Dose0.5 | -15.2 | 1.5 | -18.1 | -12.3 | <.0001 |
| Talazoparib | Capan1 | Dose1.5 - Dose0.75 | -11.9 | 1.5 | -14.8 | -9.0 | <.0001 |
| Talazoparib | Capan1 | Dose1.5 - Dose1 | -11.6 | 1.6 | -14.7 | -8.6 | <.0001 |
| Talazoparib | Capan1 | Dose1.5 - Dose1.25 | -9.0 | 1.6 | -12.1 | -5.9 | <.0001 |
| Olaparib | BxPC-3 | Dose15 - Dose7.5 | -6.1 | 1.3 | -8.5 | -3.6 | 0.0006 |
| Olaparib | BxPC-3 | Dose22.5 - Dose7.5 | -11.9 | 1.3 | -14.3 | -9.4 | <.0001 |
| Olaparib | BxPC-3 | Dose22.5 - Dose15 | -5.8 | 1.3 | -8.2 | -3.3 | 0.001 |
| Olaparib | BxPC-3 | Dose30 - Dose7.5 | -13.9 | 1.3 | -16.4 | -11.5 | <.0001 |
| Olaparib | BxPC-3 | Dose30 - Dose15 | -7.9 | 1.3 | -10.3 | -5.4 | <.0001 |
| Olaparib | BxPC-3 | Dose30 - Dose22.5 | -2.1 | 1.3 | -4.5 | 0.4 | 0.57 |
| Olaparib | BxPC-3 | Dose37.5 - Dose7.5 | -16.0 | 1.3 | -18.5 | -13.5 | <.0001 |
| Olaparib | BxPC-3 | Dose37.5 - Dose15 | -9.9 | 1.3 | -12.4 | -7.5 | <.0001 |
| Olaparib | BxPC-3 | Dose37.5 - Dose22.5 | -4.1 | 1.3 | -6.6 | -1.7 | 0.029 |
| Olaparib | BxPC-3 | Dose37.5 - Dose30 | -2.1 | 1.3 | -4.5 | 0.4 | 0.58 |
| Olaparib | BxPC-3 | Dose45 - Dose7.5 | -21.2 | 1.3 | -23.6 | -18.7 | <.0001 |
| Olaparib | BxPC-3 | Dose45 - Dose15 | -15.1 | 1.3 | -17.6 | -12.7 | <.0001 |
| Olaparib | BxPC-3 | Dose45 - Dose22.5 | -9.3 | 1.3 | -11.8 | -6.9 | <.0001 |
| Olaparib | BxPC-3 | Dose45 - Dose30 | -7.3 | 1.3 | -9.7 | -4.8 | <.0001 |
| Olaparib | BxPC-3 | Dose45 - Dose37.5 | -5.2 | 1.3 | -7.6 | -2.7 | 0.004 |
| Olaparib | PL45 | Dose15 - Dose7.5 | -1.6 | 1.9 | -5.3 | 2.0 | 0.95 |
| Olaparib | PL45 | Dose22.5 - Dose7.5 | -4.8 | 1.9 | -8.5 | -1.2 | 0.14 |
| Olaparib | PL45 | Dose22.5 - Dose15 | -3.2 | 1.8 | -6.7 | 0.3 | 0.48 |
| Olaparib | PL45 | Dose30 - Dose7.5 | -9.8 | 1.9 | -13.5 | -6.1 | 0.0002 |
| Olaparib | PL45 | Dose30 - Dose15 | -8.2 | 1.8 | -11.7 | -4.7 | 0.001 |
| Olaparib | PL45 | Dose30 - Dose22.5 | -5.0 | 1.8 | -8.5 | -1.5 | 0.09 |
| Olaparib | PL45 | Dose37.5 - Dose7.5 | -17.2 | 1.9 | -20.8 | -13.5 | <.0001 |
| Olaparib | PL45 | Dose37.5 - Dose15 | -15.6 | 1.8 | -19.0 | -12.1 | <.0001 |
| Olaparib | PL45 | Dose37.5 - Dose22.5 | -12.4 | 1.8 | -15.9 | -8.9 | <.0001 |
| Olaparib | PL45 | Dose37.5 - Dose30 | -7.4 | 1.8 | -10.9 | -3.9 | 0.003 |
| Olaparib | PL45 | Dose45 - Dose7.5 | -27.6 | 1.9 | -31.3 | -24.0 | <.0001 |
| Olaparib | PL45 | Dose45 - Dose15 | -26.0 | 1.8 | -29.5 | -22.5 | <.0001 |
| Olaparib | PL45 | Dose45 - Dose22.5 | -22.8 | 1.8 | -26.3 | -19.3 | <.0001 |
| Olaparib | PL45 | Dose45 - Dose30 | -17.8 | 1.8 | -21.3 | -14.3 | <.0001 |
| Olaparib | PL45 | Dose45 - Dose37.5 | -10.4 | 1.8 | -13.9 | -6.9 | <.0001 |
| Olaparib | Capan1 | Dose15 - Dose7.5 | -5.3 | 1.3 | -8.0 | -2.7 | 0.006 |
| Olaparib | Capan1 | Dose22.5 - Dose7.5 | -10.7 | 1.3 | -13.3 | -8.0 | <.0001 |
| Olaparib | Capan1 | Dose22.5 - Dose15 | -5.3 | 1.3 | -8.0 | -2.7 | 0.006 |
| Olaparib | Capan1 | Dose30 - Dose7.5 | -12.2 | 1.3 | -14.9 | -9.6 | <.0001 |
| Olaparib | Capan1 | Dose30 - Dose15 | -6.9 | 1.3 | -9.5 | -4.3 | 0.0003 |
| Olaparib | Capan1 | Dose30 - Dose22.5 | -1.6 | 1.3 | -4.2 | 1.1 | 0.85 |
| Olaparib | Capan1 | Dose37.5 - Dose7.5 | -14.3 | 1.3 | -17.0 | -11.7 | <.0001 |
| Olaparib | Capan1 | Dose37.5 - Dose15 | -9.0 | 1.3 | -11.6 | -6.3 | <.0001 |
| Olaparib | Capan1 | Dose37.5 - Dose22.5 | -3.7 | 1.3 | -6.3 | -1.0 | 0.1 |
| Olaparib | Capan1 | Dose37.5 - Dose30 | -2.1 | 1.3 | -4.7 | 0.5 | 0.63 |
| Olaparib | Capan1 | Dose45 - Dose7.5 | -21.0 | 1.3 | -23.6 | -18.3 | <.0001 |
| Olaparib | Capan1 | Dose45 - Dose15 | -15.6 | 1.3 | -18.3 | -13.0 | <.0001 |
| Olaparib | Capan1 | Dose45 - Dose22.5 | -10.3 | 1.3 | -13.0 | -7.7 | <.0001 |
| Olaparib | Capan1 | Dose45 - Dose30 | -8.7 | 1.3 | -11.4 | -6.1 | <.0001 |
| Olaparib | Capan1 | Dose45 - Dose37.5 | -6.6 | 1.3 | -9.3 | -4.0 | 0.0004 |
| Decitabine | BxPC-3 | Dose30 - Dose15 | -0.4 | 2.3 | -4.9 | 4.0 | 1 |
| Decitabine | BxPC-3 | Dose45 - Dose15 | -5.9 | 2.2 | -10.2 | -1.6 | 0.11 |
| Decitabine | BxPC-3 | Dose45 - Dose30 | -5.5 | 2.1 | -9.5 | -1.5 | 0.11 |
| Decitabine | BxPC-3 | Dose60 - Dose15 | -5.3 | 2.3 | -9.8 | -0.8 | 0.22 |
| Decitabine | BxPC-3 | Dose60 - Dose30 | -4.9 | 2.2 | -9.1 | -0.7 | 0.24 |
| Decitabine | BxPC-3 | Dose60 - Dose45 | 0.6 | 2.1 | -3.4 | 4.6 | 1 |
| Decitabine | BxPC-3 | Dose75 - Dose15 | -8.5 | 2.2 | -12.7 | -4.2 | 0.009 |
| Decitabine | BxPC-3 | Dose75 - Dose30 | -8.0 | 2.1 | -12.1 | -4.0 | 0.008 |
| Decitabine | BxPC-3 | Dose75 - Dose45 | -2.5 | 2.0 | -6.3 | 1.3 | 0.79 |
| Decitabine | BxPC-3 | Dose75 - Dose60 | -3.1 | 2.1 | -7.1 | 0.9 | 0.66 |
| Decitabine | BxPC-3 | Dose90 - Dose15 | -14.1 | 2.2 | -18.4 | -9.8 | <.0001 |
| Decitabine | BxPC-3 | Dose90 - Dose30 | -13.7 | 2.1 | -17.7 | -9.6 | <.0001 |
| Decitabine | BxPC-3 | Dose90 - Dose45 | -8.1 | 2.0 | -12.0 | -4.3 | 0.004 |
| Decitabine | BxPC-3 | Dose90 - Dose60 | -8.7 | 2.1 | -12.8 | -4.7 | 0.003 |
| Decitabine | BxPC-3 | Dose90 - Dose75 | -5.6 | 2.0 | -9.5 | -1.8 | 0.08 |
| Decitabine | PL45 | Dose30 - Dose15 | -2.8 | 2.0 | -6.8 | 1.2 | 0.74 |
| Decitabine | PL45 | Dose45 - Dose15 | -6.1 | 2.0 | -10.1 | -2.1 | 0.056 |
| Decitabine | PL45 | Dose45 - Dose30 | -3.3 | 2.0 | -7.3 | 0.7 | 0.58 |
| Decitabine | PL45 | Dose60 - Dose15 | -9.6 | 2.0 | -13.6 | -5.7 | 0.0007 |
| Decitabine | PL45 | Dose60 - Dose30 | -6.9 | 2.0 | -10.8 | -2.9 | 0.024 |
| Decitabine | PL45 | Dose60 - Dose45 | -3.5 | 2.0 | -7.5 | 0.4 | 0.52 |
| Decitabine | PL45 | Dose75 - Dose15 | -14.7 | 2.0 | -18.7 | -10.7 | <.0001 |
| Decitabine | PL45 | Dose75 - Dose30 | -11.9 | 2.0 | -15.9 | -7.9 | <.0001 |
| Decitabine | PL45 | Dose75 - Dose45 | -8.6 | 2.0 | -12.6 | -4.6 | 0.003 |
| Decitabine | PL45 | Dose75 - Dose60 | -5.0 | 2.0 | -9.0 | -1.1 | 0.16 |
| Decitabine | PL45 | Dose90 - Dose15 | -21.3 | 2.0 | -25.3 | -17.4 | <.0001 |
| Decitabine | PL45 | Dose90 - Dose30 | -18.5 | 2.0 | -22.5 | -14.6 | <.0001 |
| Decitabine | PL45 | Dose90 - Dose45 | -15.2 | 2.0 | -19.2 | -11.2 | <.0001 |
| Decitabine | PL45 | Dose90 - Dose60 | -11.7 | 2.0 | -15.7 | -7.7 | <.0001 |
| Decitabine | PL45 | Dose90 - Dose75 | -6.7 | 2.0 | -10.6 | -2.7 | 0.03 |
| Decitabine | Capan1 | Dose30 - Dose15 | -2.3 | 2.0 | -6.1 | 1.6 | 0.85 |
| Decitabine | Capan1 | Dose45 - Dose15 | -4.3 | 2.1 | -8.3 | -0.2 | 0.33 |
| Decitabine | Capan1 | Dose45 - Dose30 | -2.0 | 2.1 | -6.1 | 2.0 | 0.92 |
| Decitabine | Capan1 | Dose60 - Dose15 | -6.6 | 2.0 | -10.5 | -2.8 | 0.025 |
| Decitabine | Capan1 | Dose60 - Dose30 | -4.4 | 2.0 | -8.2 | -0.5 | 0.26 |
| Decitabine | Capan1 | Dose60 - Dose45 | -2.3 | 2.1 | -6.4 | 1.7m | 0.86 |
| Decitabine | Capan1 | Dose75 - Dose15 | -10.8 | 2.0 | -14.6 | -6.9 | 0.0001 |
| Decitabine | Capan1 | Dose75 - Dose30 | -8.5 | 2.0 | -12.4 | -4.7 | 0.002 |
| Decitabine | Capan1 | Dose75 - Dose45 | -6.5 | 2.1 | -10.6 | -2.5 | 0.04 |
| Decitabine | Capan1 | Dose75 - Dose60 | -4.2 | 2.0 | -8.0 | -0.3 | 0.3 |
| Decitabine | Capan1 | Dose90 - Dose15 | -22.1 | 2.0 | -25.9 | -18.3 | <.0001 |
| Decitabine | Capan1 | Dose90 - Dose30 | -19.8 | 2.0 | -23.7 | -16.0 | <.0001 |
| Decitabine | Capan1 | Dose90 - Dose45 | -17.8 | 2.1 | -21.9 | -13.8 | <.0001 |
| Decitabine | Capan1 | Dose90 - Dose60 | -15.5 | 2.0 | -19.3 | -11.6 | <.0001 |
| Decitabine | Capan1 | Dose90 - Dose75 | -11.3 | 2.0 | -15.1 | -7.5 | <.0001 |
